# Serpin 4/5 of *Nosema bombycis*: Molecular Characterization, Subcellular Localization and Pathogenic Roles in Interactions with *Bombyx mori*

**DOI:** 10.3390/microorganisms14061254

**Published:** 2026-06-02

**Authors:** Muhammad Usman Faryad Khan, Quanlin Liu, Wenxin Yang, Athumani Elias Idrisa, Jialing Bao, Maoshuang Ran, Guoqing Pan

**Affiliations:** 1 State Key Laboratory of Resource Insects, Southwest University, Chongqing 400715, China; khan_usman007@outlook.com (M.U.F.K.); l1322419056@163.com (Q.L.); 18375902457@163.com (W.Y.); athumaninyaso@gmail.com (A.E.I.); baojl@swu.edu.cn (J.B.); 2 Chongqing Key Laboratory of Microsporidia Infection and Control, Southwest University, Chongqing 400715, China; 3 Department of Biological Sciences, Dar es Salaam University College of Education, Dar es Salaam P.O. Box 2329, Tanzania

**Keywords:** hemolymph melanization, host–pathogen interaction, microsporidia effectors, secretory potential, immune evasion

## Abstract

*Nosema bombycis*, the causal agent of silkworm pébrine disease, causes substantial economic losses to sericulturists annually. Previously, 19 serpin genes (NbSPNs) were identified in this parasite, but most of their functions remain unidentified yet. Here, we provide a functional and cellular characterization of NbSPN4 and NbSPN5. Bioinformatics tools predicted four *cis*-regulatory motifs in the promoter region of NbSPN genes. A yeast signal sequence trap (YSST) assay confirmed the computationally predicted N-terminal signal peptide for NbSPN4 but not for NbSPN5. Immunofluorescence assay revealed that NbSPN4 was localized to the nucleus and NbSPN5 to the cytoplasm of infected host BmE cells. Recombinant NbSPN4/5 proteins significantly inhibited host hemolymph melanization and phenoloxidase activity in vitro, demonstrating their immune-regulatory roles. These findings provide essential insights into the roles of NbSPNs in host–pathogen interactions during *N. bombycis* infection.

## 1. Introduction

*Nosema bombycis*, a microsporidian parasite, was first identified in 1857 [1,2]. It is considered the primary cause of pébrine disease in the silkworm *Bombyx mori,* which leads to significant economic losses and damage to the sericulture industry, notably by reducing cocoon size and silkworm egg production [3]. As an obligate intracellular parasite, it can infect several silkworm larval generations, causing severe symptoms such as stunted larval growth, reduced appetite, premature death and distinctive dark brown spots on the larval cuticle [4,5,6]. The eradication of *N. bombycis* has become a major challenge for sericulturists because, in addition to horizontal transmission, the mechanism of transovarial transmission through eggs markedly enhances the parasite’s impact on the sericulture industry, necessitating the adoption of mandatory quarantine measures worldwide [7,8]. Despite the widespread use of chemicals (disinfectants) and physical control measures (microscopy and egg screening), detection and eradication have become challenging either due to delayed symptom onset or high contagion rate [9]. Recent research has focused on molecular identification, host immune evasion, and the development of additional management strategies to combat this infection [6,8,10]. Further research is necessary to examine the pathogenicity of *N. bombycis*, as it appears to be becoming increasingly pathogenic over time.

*Nosema bombycis* infection can significantly inhibit hemolymph melanization [11], immune signaling pathways, apoptosis and energy metabolism processes of the host [12,13]. Transcriptomic analysis has revealed changes in silkworm energy metabolism during *N. bombycis* infection, which upregulates host energy metabolism but maintains ATP homeostasis [14]. These abnormal physiological changes in the host are precisely regulated by effector molecules secreted by the pathogen, which disrupt the host immune defense system, modulate host cell physiology and create favorable conditions for its invasion, proliferation and transmission [15]. Effector proteins are the key molecules that mediate interactions between intracellular pathogens and their hosts. In *N. bombycis*, several secreted effector proteins have been identified, such as hexokinase, the Ricin-B-lectin protein family and the serine protease inhibitor (Serpin) family, which are actively involved in host immune evasion and metabolic reprogramming [16,17,18].

Serine Protease Inhibitors constitute a diverse superfamily of protease inhibitors found across all living organisms, including plants and animals [19,20]. They interact with target proteases to form complexes, regulating various biological processes including blood homeostasis [21], inflammatory responses [22] and cell apoptosis [23]. Blood-feeding parasites, e.g., ticks, express serpins such as Hl Serpin-ac and Hl Serpin-b, which inhibit host proteases, including cathepsin G and coagulation factor Xa, thereby delaying immune responses and inflammation [24]. To date, 53 serpin genes have been identified in the genus *Nosema*, of which 21 are predicted to contain signal peptides (SPs) [14]. Structural analysis has shown that serpins are characterized by a common core domain composed of three β-sheets and 8–9 α-helices along with the reactive center loop (RCL) [25] and also their primary mechanism of action involves the RCL, which undergoes a conformational change that maintains thermodynamic stability and facilitates the reaction [26]. In *N. bombycis*, 19 serpin genes (NbSPNs) were recently identified [14]. Some of them play a crucial role in suppressing melanization, phenoloxidase (PO) activity and host apoptosis, such as NbSPN6 localizes in the host cytosol, leading to inhibit melanin formation and suppress PO activity by targeting the PPAE enzyme in the host [11] and NbSPN14 which was initially secreted in the host cytosol during the early stages of infection and subsequently translocated into the nucleus exhibiting anti-apoptotic activity by inhibiting host caspases via BmICE enzyme [13].

These distinct patterns of NbSPNs encouraged us to further investigate their roles and characteristics, which remain unexplored yet. Therefore, NbSPN4 and NbSPN5 were selected based on bioinformatics analysis, promoter region sequence similarities, distinct secretion patterns, subcellular localization and Hemolymph melanization inhibition assay. We found that NbSPN4 with a canonical SP resides in the host nucleus, whereas NbSPN5 without an SP distributes in the cytoplasm. Both NbSPN4 and NbSPN5 proteins effectively inhibit host hemolymph melanization and PO activity. This work provides new clues for uncovering their interacting partners and further illustrating the pathogenic mechanism of *N. bombycis*.

## 2. Materials and Methods

### 2.1. Promoter Region Analysis of NbSPN Family

Promoter region sequences of the *N. bombycis* serpin gene family (NbSPN1–NbSPN19) were extracted up to 500 bp upstream of each gene’s start codon from the National Center for Biotechnology Information (NCBI) and Microsporidia Database (https://microsporidiadb.org/micro/app, accessed on 24 May 2024). All promoter sequences on the negative strand were reverse-complemented, whereas those on the forward strand were left unchanged. Additionally, sequences were screened to exclude any overlaps with upstream coding regions. De novo motif discovery was conducted by MEME suite (v5.x) under the zero or one occurrence per sequence (ZOOPS) model, with motif widths limited to 5–20 bp and a maximum of four statistically significant motifs. Motifs were represented as position weight matrices (PWMs), and logos were generated to observe nucleotide conservation within the motifs [27,28]. Identified motifs were compared against a fungal cis-element database (JASPAR Fungi 2024) using TOMTOM v 5.5.9 (accessed on 8 June 2025) and the best-scoring matches were selected [29] to evaluate the motif location and conservation of the identified motifs. Individual promoter sequences were scanned with FIMO at a significance threshold of *p* < 1 × 10^−4^ and a false discovery rate (FDR) correction was applied to obtain q-values. The distribution of motifs within the promoter regions was visualized by mapping the motif coordinates relative to the start codon [30,31].

### 2.2. Silkworm Rearing, Spore Extraction and BmE Cell Infection

The silkworm Dazao strain was acquired from the Gene Resource Library of Domesticated Silkworms (Southwest University, Chongqing, China). Fresh mulberry leaves were rinsed with sterile distil water, air-dried and fed to larvae every 6 h in a controlled environment with 70% ± 5% relative humidity at 25 ± 1 °C and a 12 h light/dark cycle. To infect silkworms, a small batch of mature spores of *N. bombycis* Chongqing 1 (CQ 1) was obtained from the State Key Laboratory of Resource Insects, Southwest University, China. The spores were then suspended at 10^6^/mL, evenly spread onto fresh mulberry leaves and fed to the silkworm larvae to induce infection. The spores used in this study were isolated from infected silkworm pupae and purified by Percoll density gradient centrifugation with slight modifications [32]. To infect BmE cells, the purified spores underwent a 3 min treatment with 0.1 M KOH at a 10:1 ratio in Grace’s medium at 28 °C. After a 2 h incubation period, the medium was refreshed, and the cells were cultured in a T-25 flask at 28 °C in Grace’s medium, which was supplemented with 10% (V/V) fetal bovine serum (FBS) from Gibco and 1% (V/V) penicillin-streptomycin [33].

### 2.3. Bioinformatic Analysis

To predict molecular weight and isoelectric point (pI) ExPASy (ProtParam) was used and TargetP 2.0 for subcellular localization, servers were accessed on 4 September 2025. The AI-based three-dimensional (3D) structures of NbSPN4 and NbSPN5 (NCBI acces-sion no. EOB14654.1 and EOB14656.1, respectively) were predicted by AlphaFold2 online tool (https://colab.research.google.com/github/sokrypton/ColabFold/blob/main/AphaFold2.ipynb, accessed on 3 April 2025) with default settings. The predicted 3D models were further validated by Ramachandran plot analysis and visualized by PyMOL v1.7. The secondary structure was analysed with PDBsum, which was used to assign α-helices, β-strands, and turns from the coordinate data. The resulting topology and residue-level annotations were then used for comparative structural mapping. Lastly, the P1 site was predicted using a previously defined protocol [11].

### 2.4. Signal Peptide Prediction and Validation

SignalP 5.0 (http://www.cbs.dtu.dk/services/SignalP/, accessed on 8 March 2025) was used to predict SP, and the yeast signal sequence trap system was employed to confirm the computationally predicted SP. PCR Primers were designed using NotI and XhoI restriction enzymes to create in-frame fusions with the invertase DNA fragments extracted from cDNA of NbSPN4 and NbSPN5, which encode the SP sequence of both genes. The vector pSUC2T7M13ORI was used to construct NbSPN4-SP-pSUC2 and NbSPN5-SP-pSUC2 using the LiAc method [34]. It contains the invertase gene SUC2, which lacks both the transcription-initiating Met and SP. Additionally, the invertase-negative yeast strain YTK12 was transformed into a pSUC2 derived plasmid individually using the lithium acetate (LiAc) method (PS87 and Mg87 were used as positive and negative controls, respectively). The yeast was plated on CMD-W (minus Trp) plates, containing 0.67% yeast N-base without amino acids (0.075% dropout supplement, 2% sucrose, 0.1% glucose and 2% agar A). Transformed colonies were further plated onto fresh CMD-W plates and incubated at 30 °C for 2–3 days. The transformation success rate was determined by colony PCR with vector-specific universal primers. To identify invertase secretion, colonies were replicated onto YPRAA plates (1% yeast extract, 2% peptone, 2% raffinose, 0.002% Antimycin A and 2% agar A), which contained raffinose but lacked glucose. Another method, which converts 2,3,5-Triphenyltetrazolium Chloride (TTC) into insoluble red triphenyl formazan, was used to measure invertase enzymatic activity. The yeast transformants were added to 5 mL of CMD-W medium (the YTK12 non-transformed strains were cultivated in YPDA medium) and incubated for 36 h at 30 °C and 200 rpm. The media was centrifuged to obtain a pellet, washed with sterile distilled water and incubated with 2 mL 0.1% TTC for 35 min at 35 °C, followed by 5 min at room temperature. Finally, a color shift was observed in the samples.

### 2.5. Antigen Preparation and Antibody Production

Antigens were prepared from the predicted epitopes of both genes using Bepipred (https://tools.iedb.org/bcell/, accessed on 24 May 2024). The first 224 amino acids (672 bp), excluding the signal peptide (SP, residues 1–27) and containing 71 predicted epitopes, were selected for NbSPN4-Epi-pET28a vector construction. Similarly, a 194-amino-acid fragment (582 bp) containing 45 predicted epitopes was selected for NbSPN5-Epi-pET28a. Primers were designed to amplify these cDNAs using BamHI and HindIII restriction sites (Table 1), and the resulting fragments were cloned into the pET28a vector for protein extraction and purification. Subsequently, 100 μL of purified recombinant protein, adjusted to 1 mg/mL, was emulsified 1:1 with complete Freund’s adjuvant (Sigma-Aldrich, St. Louis, MO, USA) until a white emulsion was formed. The resulting emulsion was administered to BALB/c mice subcutaneously at 2 different sites. After 2 weeks, booster doses were administered in the same manner using incomplete Freund’s adjuvant at 1-week intervals for three additional weeks. The mice were anesthetized, and blood was collected after the final immunization via retro-orbital bleeding. The sera containing anti-NbSPN4 and anti-NbSPN5 were incubated at 37 °C for 1 h, then at 4 °C for 4 h, and the samples were centrifuged at 4 °C for 15 min at 5000 rpm. Plasma was collected, verified by Western blotting against antigenic and total protein of Nb-infected BmE cells then stored at −20 °C.

### 2.6. Western Blot

To validate the expression of NbSPN4 and NbSPN5 in infected BmE cells, a Western blot assay was performed. The cell lysate of *N. bombycis* from infected BmE cells was used with 300 μL of RIPA lysis buffer (Beyotime, Shanghai, China) supplemented with 1 mM PMSF. The lysates were incubated on ice for 15 min and clarified by centrifugation at 12,000× *g* for 15 min at 4 °C. The supernatant was collected and protein samples were separated by 10% SDS-PAGE followed by transfer onto a polyvinylidene difluoride (PVDF) membrane (Roche, Basel, Switzerland). The membrane was blocked in TBST buffer (10 mM Tris-HCl, 150 mM NaCl and 0.1% Tween-20 with pH 7.4) containing 5% (*w*/*v*) skim milk for 1 h at room temperature. After blocking, the membrane was incubated with primary antibodies against NbSPN4 or NbSPN5 for 2 h at room temperature with shaking, the membrane was washed three times with TBST buffer and incubated with a goat anti-mouse IgG secondary antibody (Thermo Fisher, Waltham, MA, USA) for 50 min at room temperature washed again and finally the protein bands were visualized using the Clarity Western ECL substrate (Bio-Rad, Hercules, CA, USA) imaged with a chemiluminescent imaging system.

### 2.7. Indirect Immunofluorescence Assay (IFA)

To investigate the sub-cellular localization of NbSPN4 and NbSPN5 during *N. bombycis* infection, the infected cells were harvested, fixed with 4% paraformaldehyde for 10 min at room temperature, followed by three washes with PBS. The fixed cells were then permeabilized with 0.1% Triton X-100 for 15 min, followed by incubation in PBST (PBS containing 0.1% Tween 20) supplemented with 5% Bovine Serum Albumin (BSA) and 10% goat serum for 1 h. After blocking, the cells were washed three times with PBST, incubated with anti-NbSPN4 and anti-NbSPN5 separately, followed by anti-Nb spores for 2 h at room temperature and then washed three times with PBST again. The cells were finally incubated with Alexa Fluor 594 conjugated with goat anti-mouse IgG and Alexa Fluor 488 conjugated with goat anti-rabbit IgG secondary antibodies (1:1000 dilution, Invitrogen, Waltham, MA, USA). The cell’s nuclei were counterstained with DAPI (1:1000, Sigma Aldrich, St. Louis, MO, USA) for 15 min at room temperature. After final washing 3–6 times with PBST, samples were mounted with an antifade mounting medium and visualized by Olympus FV1200 confocal laser scanning microscope (CLSM) equipped with a 100× oil-immersion objective, with a 5 μm scale bar. The fluorescence images were captured using Olympus FV10-ASW v 4.2 software.

### 2.8. Recombinant Protein Extraction

Recombinant NbSPN4 and NbSPN5 proteins were expressed using the Protein Factory kit (Kangma Biotechnology Co., Ltd., Shanghai, China). The ORF sequences of Nbserpin4 and Nbserpin5, together with the backbone of the pD2P_1.08t-8His-eGFP vector, were amplified by PCR (primers are listed in Table 1. All primers were self-designed and synthesized by Sangon Biotech (Shanghai) Co., Ltd., Shanghai, China). The GFP sequence in the pD2P_1.08t-8His-eGFP vector was then replaced by homologous recombination to generate the recombinant constructs.

The constructed pD2P-NbSPNs recombinant plasmids and the empty vector plasmid were used as templates and added to the DNA Amplifier at a final concentration of 1 ng/µL. The reaction mixture was incubated at 37 °C for 2 h to obtain amplified DNA, which was then added to the Protein Factory reaction at a 1:30 volume ratio for target protein expression, with a total reaction volume of 800 µL. The recombinant proteins were purified by HisSep Ni-NTA magnetic beads (Cat. No. 20561ES08; Yeasen Biotechnology, Shanghai, China). The Protein Factory reaction mixture was centrifuged at 4 °C and 4000 rpm for 3 min, and the supernatant was collected. Twenty microliters of His-Monster beads were washed twice with Binding Buffer. The supernatant containing the recombinant protein was then mixed with the His-Monster beads, vortexed thoroughly for 30 s, and incubated at 4 °C with rotary mixing for 1 h. The beads were collected magnetically and the supernatant was discarded. Next, 1000 µL of Washing Buffer was added, the mixture was vortexed thoroughly for 30 s, and the beads were collected magnetically again before discarding the supernatant. This washing step was repeated three times. Finally, 30–50 µL of Elution Buffer was added to the beads, mixed thoroughly by pipetting, and allowed to stand for 1 min. The beads were then magnetically separated, and the eluted supernatant was collected as the target protein. The purified recombinant proteins were analyzed by SDS-PAGE, and protein concentrations were determined by the Bradford assay with BSA as the standard. The successful expression of recombinant NbSPN4 and NbSPN5 was validated by Western blot using an anti-His tag antibody.

### 2.9. Hemolymph Melanization and PO Activity Assay

Hemolymph from 5th instar silkworm larvae was collected in a pre-chilled 1.5 mL centrifuge tube and immediately placed on ice. It was then centrifuged at 1600× *g* for 5 min to remove hemocytes. To perform the PO activity and hemolymph melanization inhibition assay, freshly prepared L-DOPA (3 mM) substrate in PBS (pH 7.4) was added to a 96-well microtiter plate, followed by 1 μg recombinant protein, GFP protein and hemocyte-free hemolymph. The reaction mixture was incubated at room temperature, and absorbance at 492 nm was measured every 5 min for up to 60 min using a microplate reader. The dopachrome formation in the 96-well plate was analyzed morphologically and it revealed that inhibition of melanin synthesis was evident. The PO activity was calculated according to the following formula [11].

PO activity (U/mL) = 0.001 × Δ492/min(1)

Simultaneously, photos were taken at the start, middle, and end of the reaction to observe any significant differences in hemolymph melanization rates between the control and experimental groups. Phenylthiourea (PTU) was used as a positive control. Two-way ANOVA with Dunnett’s test was used for statistical analysis. All samples were used in triplicate.

### 2.10. Statistical Analysis

All quantitative experiments were performed in four biological replicates. Two-way analysis of variance (ANOVA) followed by Dunnett’s multiple comparison test was adopted for statistical comparison. Statistical significance was defined as * *p* < 0.05, ** *p* < 0.01, *** *p* < 0.001 and **** *p* < 0.0001.

## 3. Results

### 3.1. Cis-Elements Prediction of N. bombycis Serpin Gene Family

**Figure 1:**
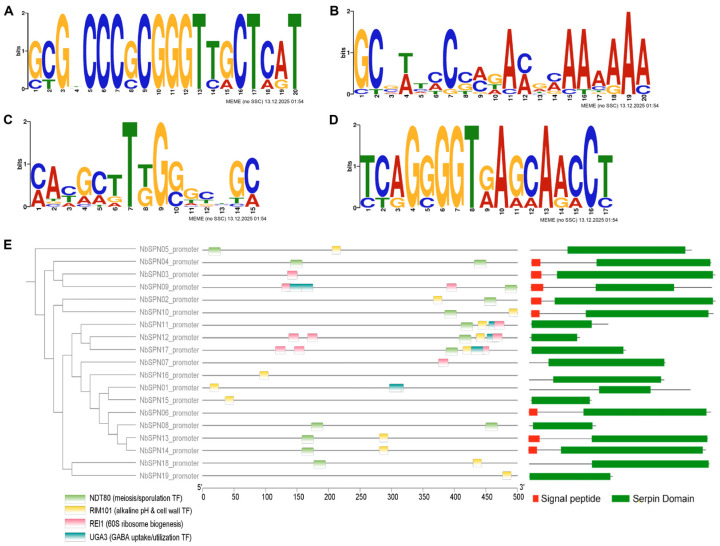
Predicted motifs and promoter comparison of the NbSPN gene family. (**A**) Motif 1 contains the conserved CCC homopolymer core present in various *Nosema* species and resembles UGA3 TF with eight sites and 20.83% presence in the promoter region of the *NbSPN*s gene family. (**B**) Motif 2 resembles RIM101 with 13 sites and 27.08% similarity. (**C**) Motif 3 mimics NDT80, the most prevalent TF with 14 sites (29.17%) and regulates the sporulation phase. (**D**) Motif 4 resembles REI1, with 11 sites and a 22.92% share across the promoter regions of NbSPNs. (**E**) Represents the conservation of TFs and their locations across the NbSPN gene family. Motifs were discovered by MEME-Suite (Zoops setting with a 5–20 bp motif width), annotated by TOMTOM, and scanned/positioned with FIMO (q ≤ 0.05). Elements were visualized using TBtools-II v 2.388 Basic Bio Sequence Viewer. The signal peptide (red) and serpin domain (green) predictions are presented on the right.

In microsporidia, the regulatory motifs at transcript initiation sites appear to be concealed within a short *cis*-acting regulatory region upstream of the gene, a consequence of their compact and gene-dense genomes [35,36]. To gain insights into the potential *cis*-regulatory control of *N. bombycis*, the *NbSPN* gene family was analyzed, which predicted four conserved motifs across the serpin gene family (Figure 1A–D). The motifs were further characterized to assess their resemblance to *cis*-regulatory elements (Transcription factor binding sites, TFs) by using the fungal-domain database JASPAR Fungi 2024 [29], and their locations were explored and mapped across all NbSPNs (Figure 1E). The *cis*-element map revealed a distinct and conserved distribution of *cis*-elements across all NbSPNs, suggesting that they may regulate serpin gene expression.

#### Functional Exploration of Cis-Elements

In total, 48 predicted *cis*-elements were identified across all NbSPN promoter sequences. Among these, NDT80 was the most prevalent, accounting for 29.17% of all sites (14 sites), resembling motif C in yeast, whereas RIM101 resembled motif B 27.08% (13 sites) and is a zinc-finger like TF in *Candida albicans* that induces an alkaline response [37], followed by REI1 and UGA3, which mimic motifs D and A (22.92%, 11 sites and 20.83%, 10 sites respectively). Interestingly, two NDT80 TFs in NbSPN4, one NDT80 and one RIM101, each among the NbSPN5 promoter sequences, were conserved and predicted to regulate sporulation and stress response in yeast [38,39,40]. In *N. bombycis,* these elements may be involved in regulating gene expression during the sporulation phase and in responses to external stresses to the parasite (such as alkaline pH and host immune response).

### 3.2. Characteristics of NbSPN4 and NbSPN5

**Figure 2:**
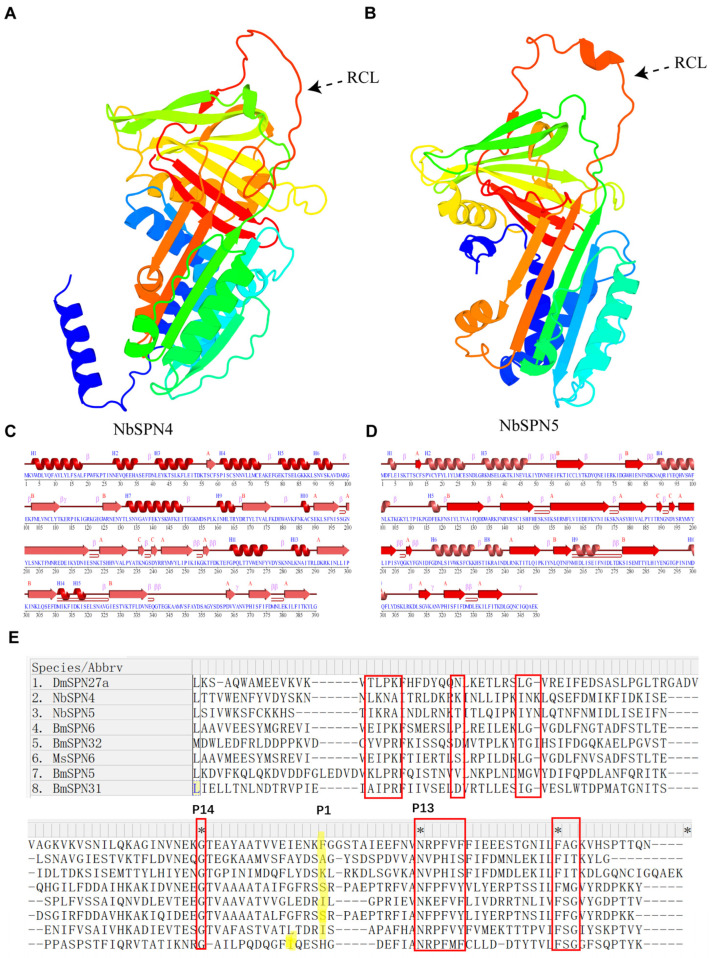
Structural and sequence analysis of NbSPN4, NbSPN5 and P1 site prediction. (**A**,**B**) Three-dimensional ribbon structures of NbSPN4 and NbSPN5 generated by AlphaFold showing the typical serpin domain architecture with 3 β-sheets, 8–9 α-helices and the RCL indicated by arrows. (**C**,**D**) Secondary structure predictions of NbSPN4 (**C**) and NbSPN5 (**D**) reveal conserved α-helices and β-strands consistent with the serpin scaffold. (**E**) Multiple sequence alignment of NbSPN4 and NbSPN5 was performed using MEGA 11.0. Red brackets indicate conserved motifs and asterisks (*) denote 100% conserved amino acids across all organisms. The predicted P1 site is highlighted in yellow, primarily based on differences between the P14 and P1 positions, including gaps. Both NbSPN4 and NbSPN5 were predicted to have serine (S) at the P1 site. Dm refers to *Drosophila melanogaster*, Bm to *B. mori*, Ms to *Manduca sexta*, Nb to *N. bombycis*, and SPN to serpin.

In silico analysis predicted that NbSPN4 comprises 390 amino acids (aa) and has a molecular weight of 45.0 kDa with a theoretical pI of 8.62, whereas NbSPN5 has 350 aa, a molecular weight of 41.2 kDa and a pI of 7.97. Notably, TMHMM 2.0 analysis showed no predicted transmembrane helices for NbSPN4 and NbSPN5 (score < 0 TMHs; first 60 residues), suggesting that neither is a transmembrane protein (Figure S1A,B). The predicted 3D models of NbSPN4 and NbSPN5 indicate that both adopted a conserved core of three β-sheets surrounded by 8–9 α-helices with an exposed reactive center loop (RCL) protruding outside the structure (Figure 2A,B). The secondary structure (Figure 2C,D) further supports this interpretation, showing a broadly conserved pattern of α-helices and strands across both proteins that matches the expected serpin topology with differences localized mainly to the RCL regions rather than the structured core.

Moreover, serine (S) aa was predicted as the P1 site in the RCL region of both proteins based on the differences primarily between the P14 and P1 sites (Figure 2E), which predicts that they may share a common inhibitory mechanism but may differ in target protease preference due to differences in RCL sequence and conformation.

### 3.3. NbSPN4 and NbSPN5 SP Prediction and Validation Using YSST Assay

**Figure 3:**
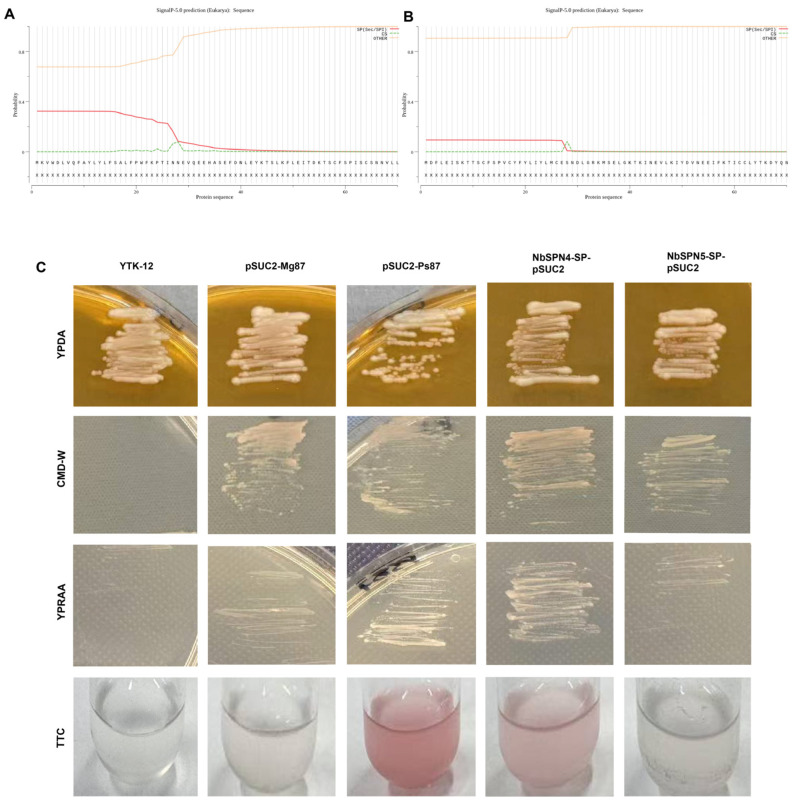
In silico SP prediction and experimental verification by employing the YSST assay. (**A**) SignalP 5.0 analysis of NbSPN4 predicted a secreted signal peptide. (**B**) SignalP 5.0 analysis of NbSPN5 suggested no signal peptide. (**C**) The *S. cerevisiae* YTK12 strain carrying the pSUC2 plasmid fused with the SP sequences of NbSPN4 and NbSPN5 was able to grow on CMD-W selective media (yeast is dependent on sucrose in the absence of invertase secretion), whereas on YPRAA (media containing raffinose instead of sucrose, and strains only grow when invertase is secreted), reduction of TTC to formazan produced a red color, indicating secretion of invertase (functional SP and no color shift indicate non-functional or no SP, with Mg87 and Ps87 serving as negative and positive controls, respectively).

The SignalP 5.0 predicted that NbSPN4 has an N-terminal SP of 1–27 aa (Figure 3A), suggesting that it may be secreted, whereas NbSPN5 lacks this feature (Figure 3B). To verify this prediction, we performed the YSST assay. The NbSPN4-SP-pSUC2 and NbSPN5-SP-pSUC2 plasmids were constructed and transformed into the YTK12 strain of *Saccharomyces cerevisiae*. In CMD-W medium, transformants carrying pSUC2 grew due to the nutrient deficiency compensated by the tryptophan operon, whereas wild-type YTK12 did not survive, validating the vector’s functionality. By transforming the SP sequence into pSUC2, the plasmid restored sucrase secretion, allowing SP-positive strains to grow normally on YPRAA medium. Screening on YPRAA media revealed that only strains with a functional SP grew on YPRAA media by decomposing raffinose and utilizing it as a carbon source, whereas non-functional or no SP strains did not grow on YPRAA. We further verified our results by TTC colorimetric assay. Strains carrying functional SPs (NbSPN4) were able to secrete sucrase, which hydrolyzed sucrose to produce reducing sugars, which then reacted with TTC to form a red color, whereas non-functional SP-carrying strains were unable to hydrolyze sucrose, hence, no color shift was observed. The results were consistent with the positive and negative controls, Ps87 and Mg87, respectively (Figure 3C). In summary, the SP sequence NbSPN4 was functional and could guide extracellular protein secretion, whereas NbSPN5 showed no effect, consistent with the signal peptide prediction.

### 3.4. Recombinant Protein Extraction, Antibody Production and Immunoblotting

**Figure 4:**
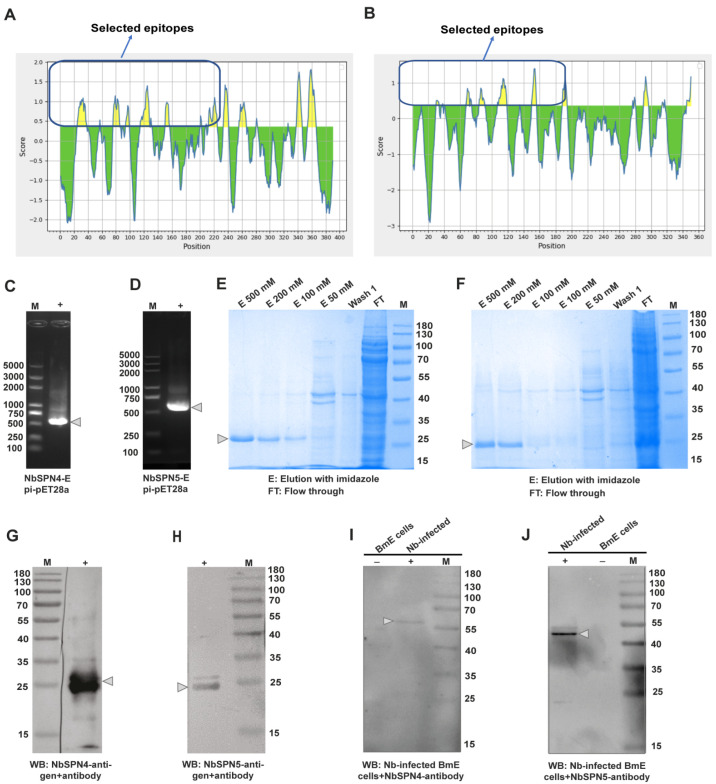
Epitope prediction, antigen preparation and immunoblotting analysis. (**A**,**B**) B-cell epitopes of NbSPN4 and NbSPN5 were predicted by IEDB 1.0 and the cDNA of 1st 672 bp post SP sequence (61–732 bp) of NbSPN4 and 594 bp of NbSPN5 (1–594 bp) highlighted in a blue box only were considered for antigen preparation. (**C**) Represents a NbSPN4-Epi-pET28a agarose gel PCR band of interest (**D**). Shows NbSPN5-Epi-pET28a agarose gel PCR band of interest (**E**). NbSPN4-Epi-pET28a antigenic protein extraction and confirmation by SDS-PAGE, which shows an expected band of antigen indicated by arrowheads, and M is a marker of 180 kDa. (**F**) NbSPN5-Epi-pET28a antigenic protein extraction and confirmation by SDS-PAGE, which shows an expected band of antigen indicated by arrowheads, and M is a marker of 180 kDa. (**G**) Western blot confirmation of the anti-NbSPN4 antibody against the antigen administered to mice, the arrowheads indicate the expected band of the antigen and M is a marker of 180 kDa. (**H**) Western blot confirmation of anti-NbSPN5 antibody antigenic band is indicated by arrowheads, and M is a marker of 180 kDa. (**I**,**J**) The antibodies were further confirmed against total protein of *N. bombycis* infected and uninfected BmE cells, where plus (+) sign indicates the detection of expected bands of both NbSPN4 and NbSPN5 in the lanes carrying total protein of infected BmE cells and uninfected BmE cells lanes remain negative (−) did not show any band which confirms the sensitivity of antibodies, where M is the marker of 180 kDa and bands of interest indicated by arrowheads.

Antibodies against both proteins were produced through careful epitope selection (Threshold > 0.35, Figure 4A,B). The cDNA of 672 bp post SP (61–732 bp) of NbSPN4 and 594 bp of NbSPN5 (1–594 bp) Figure 4C,D) were selected, confirmed via Sanger sequencing, cloned into DH5α cells and transformed into BL-21 (DE3) cells. Antigenic proteins of approximately 25 kDa with selected epitopes were extracted (Figure 4E,F) and administered to mice to challenge their immune systems and elicit antibodies against the foreign antigen. After 5 weeks of immunization, total blood was collected via retro-orbital bleeding, and the collected blood serum was confirmed against the antigenic protein via Western blot (Figure 4G,H), further confirmation was performed against total protein of infected BmE cells (Figure 4I,J), which proved that both antibodies strongly recognized the target bands in the antigenic proteins as well as in the total protein extracts of *N. bombycis*-infected BmE cells.

### 3.5. Subcellular Localization of NbSPN4 and NbSPN5 in Infected BmE Cells

**Figure 5:**
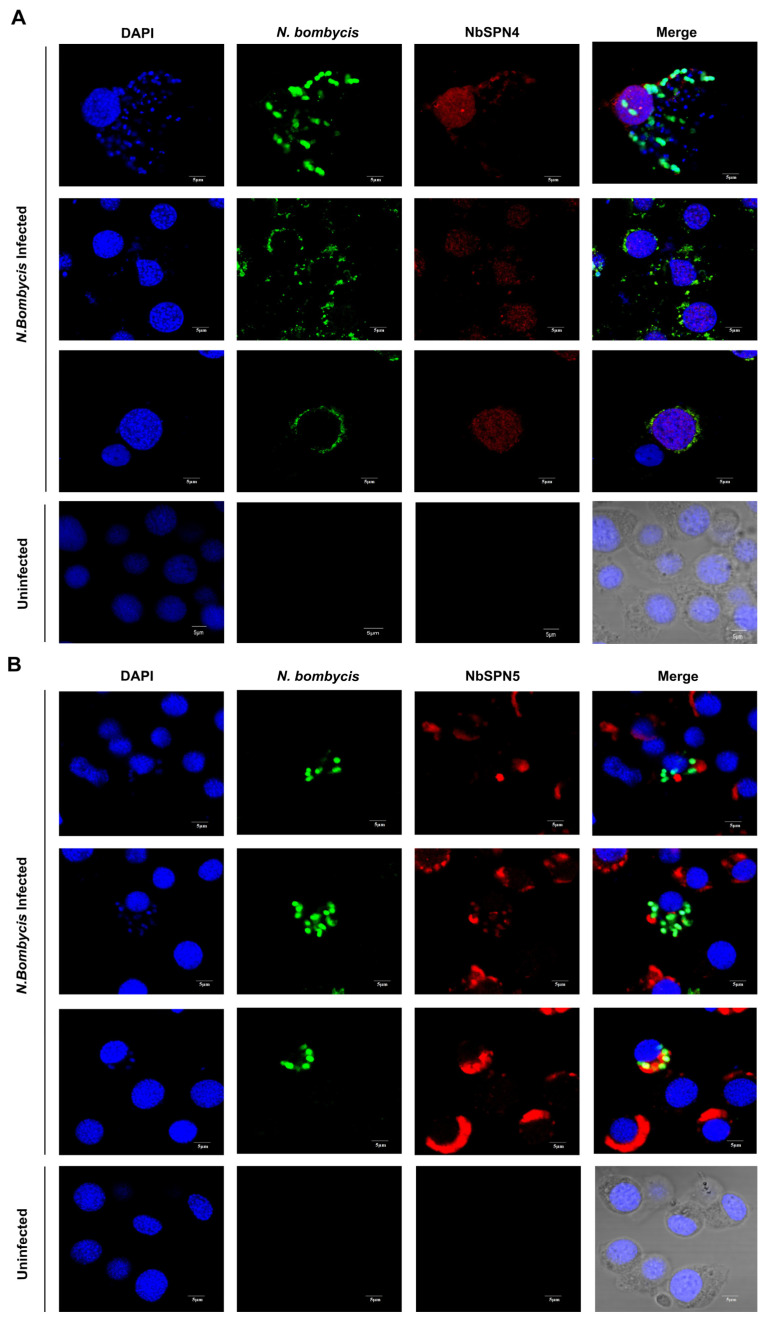
Subcellular localization of NbSPN4 and NbSPN5 in infected BmE cells. An immunofluorescence assay was conducted using antibodies against *N. bombycis* (Anti-Nb, green), NbSPN4 or NbSPN5 (red), with nuclei counterstained with DAPI (blue). (**A**) CLSM imaging revealed NbSPN4 localization in the nucleus of infected host BmE cells throughout the *N. bombycis* infected panel. In contrast, the *N. bombycis* uninfected panel served as a negative control representing no detectable signals for either NbSPNs or *N. bombycis*. The nuclei of the cells were stained with DAPI blue, *N. bombycis* labelled with rabbit-anti-spore antibody and a goat-anti-rabbit secondary antibody conjugated with Alexa fluor 488 (green), NbSPN4 with mouse polyclonal anti-NbSPN4 antibody and a goat anti-mouse secondary antibody conjugated with Alexa fluor 594 (red). (**B**) The Subcellular localization of NbSPN5 was assessed using the same protocol, with the exception that the anti-NbSPN5 antibody and a goat anti-mouse secondary antibody conjugated with Alexa Fluor 594 (red) were used instead of anti-NbSPN4, which revealed cytoplasmic subcellular localization of NbSPN5 in host BmE cells. Merged panels illustrate the overlay of signals alongside bright-field images. Scale bar 5 μm.

To determine the subcellular localization of NbSPN4 and NbSPN5 in host cells, we performed an indirect immunofluorescence assay (IFA) in BmE cells at 48 h post infection (hpi). The NbSPN4 immunostaining (red) was primarily concentrated within the DAPI-stained nuclear region across the infected cell panels. In contrast, the uninfected control panels showed DAPI-stained nuclei but no detectable green (parasite) or red (NbSPN4) fluorescence, confirming the specificity of staining and a low background (Figure 5A). By incorporating the same IFA workflow, NbSPN5 immunostaining (red) was mainly distributed in the cytoplasm surrounding the DAPI-stained nuclei in infected cells (Figure 5B). Overall, these results indicate distinct subcellular localizations in host BmE cells, NbSPN4 was primarily localized in the nucleus, whereas NbSPN5 was confined to the cytoplasm during *N. bombycis* infection.

### 3.6. NbSPN4 and NbSPN5 Inhibited Host Hemolymph Melanization and PO Activity

**Figure 6:**
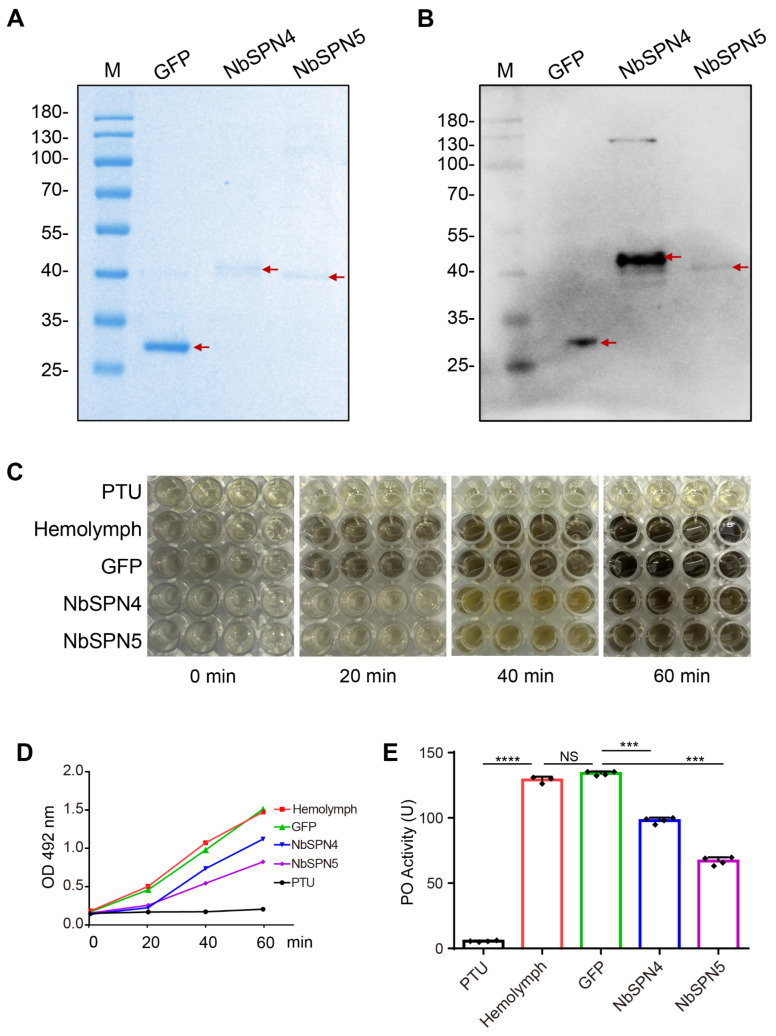
Hemolymph melanization and PO activity assay by recombinant NbSPN4 and NbSPN5. (**A**) Recombinant NbSPN4 and NbSPN5 proteins were expressed and purified in vitro using the Protein Factory kit, with GFP protein as the control. Red arrows indicate target protein bands. (**B**) Western blot verification of recombinant NbSPN4 and NbSPN5 proteins using His antibody, with GFP as the control. Red arrows indicate target protein bands. (**C**) The visual display of dopachrome formation in 96-well plates at 0, 20, 40, and 60 min showed reduced melanin production in serpin-treated samples compared to the hemolymph as a negative control and GFP protein, PTU was used as the positive control. (**D**) Represents OD 492 nm absorbance changes overtime, which identifies that the recombinant proteins inhibited melanization as compared to the untreated hemolymph, and PTU was used as the positive control and GFP as the negative control. (**E**) Bar graph illustrates PO activity. Hemolymph treated with recombinant NbSPN4 and NbSPN5 showed a significant decrease in PO activity relative to the untreated control in vitro, whereas PTU served as a positive control and GFP as the negative control throughout the reaction. The final molar concentration of recombinant protein in the incubation system was approximately 100 nM. A two-way ANOVA with Dunnett’s multiple tests was applied to calculate the results (*** *p* < 0.001, **** *p* < 0.0001, all samples were measured in triplicate).

To understand how NbSPN4 and NbSPN5 affect silkworm hemolymph melanization and phenoloxidase (PO) activity, both recombinant proteins were extracted (Figure 6A,B), the purified GFP protein was used as a control to analyse its influence on the overall reaction. Hemolymph treated with NbSPN4 showed darker pigmentation compared to NbSPN5, which reflected visibly lighter pigmentation compared to the untreated normal hemolymph and GFP (Figure 6C). Melanization was quantified by measuring the absorbance at 492 nm, and the results revealed that treatment with NbSPN4 and NbSPN5 significantly inhibited the melanization process (Figure 6D). This inhibitory mechanism was initiated by a serine protease-activation cascade, which leads to PO activation and melanin formation. Therefore, PO activity in hemolymph serves as a marker of melanization and activation of the serine protease cascade. We further measured PO activity at 60 min after the reaction was initiated with L-DOPA substrate. The results demonstrated that NbSPN4 and NbSPN5 significantly suppressed PO activity, whereas GFP showed no significant inhibitory effect (Figure 6E). These results were consistent with our prior findings of NbSPN6 [11]. Thus, we can say that NbSPN4 and NbSPN5 likely play essential roles in modulating host immunity and may directly or indirectly regulate serine proteases involved in the PO activation cascade within host hemocytes.

## 4. Discussion

Serpin are crucial for biological processes and host–pathogen interactions. In contrast, very few studies have focused on pathogen-derived serpins that inhibit host immunity, such as cowpox virus CrmA, which blocks host cell apoptosis and immune responses [41]. In microsporidia, serpin genes were exclusively present in the genus *Nosema*, suggesting that *N. bombycis* serpin genes cluster with poxvirus serpin genes, indicating an evolutionary relationship with poxviruses that may have contributed to their immune-modulatory roles [14]. In this study, we analyzed the promoter regions of the *N. bombycis* serpin family and experimentally characterized NbSPN4 and NbSPN5, including SP verification, subcellular localization, and their possible effects on melanization and PO activity.

Four types of conserved cis-elements, namely NDT80, RIM101, REI1 and UGA3, were identified in the promoter regions of 19 NbSPN genes in *N. bombycis* [29]. The enriched RIM101 and REI1 elements regulate sporulation and stress responses in fungi [38,39], implying that they mediate the specific expression of NbSPNs during *N. bombycis* development, host immune challenge and environmental stress. The functions of the other two elements remain unclear. Distinct differences were observed in the composition and arrangement of promoter motifs among NbSPN members, with the copy number variation of NDT80 elements between NbSPN4 and NbSPN5 being the most prominent. This indicates that the NbSPN family possesses divergent cis-regulatory systems rather than a uniform promoter regulation pattern. Previous studies have demonstrated that NbSPN transcription is gene-specific and infection-stage dependent [14]. Collectively, variations in promoter motifs underpin the differential transcription and context-specific activation of NbSPNs. This also constitutes a key molecular mechanism whereby *N. bombycis* modulates serpin expression to adapt to host immunity and ensure successful infection and survival.

Structural modelling confirms that NbSPN4 and NbSPN5 adopt conserved serpin architecture (Figure 2A,B), which underpins the metastable state and suicide-substrate inhibition of inhibitory serpins [42,43]. Importantly, P1 site and its surrounding RCL sequences largely dictate which protease(s) will be captured, while subtle local changes can markedly disrupt target preferences even when the overall serpin scaffold is unchanged [44,45]. For both proteins, serine (S) was predicted to be at the P1 site in the RCL region (Figure 2E), but the observed divergence around the scissile-bond neighborhood and across the RCL in the secondary structure interpretation was biologically meaningful because it can shift protease recognition and inhibitory efficiency without disrupting the conserved serpin scaffold [44]. Generally, pathogen-derived serpins commonly evolve by conserving inhibitory kinetics while diversifying the RCL to counter distinct host proteases and immune pathways [46,47]. We can say that NbSPN4 and NbSPN5 may share a common core inhibitory mechanism while having functionally altered targets as a result of RCL micro-variation, enabling context-dependent interference with different host proteolytic environments during infection.

Analysis using recombinant proteins revealed that both NbSPN4 and NbSPN5 significantly inhibit hemolymph melanization and PO activity, thereby inhibiting host immune defense (Figure 6E). This finding is consistent with our previous in vivo study of NbSPN6 in silkworms [11]. Hesp018, a serine protease inhibitor encoded by Hemileuca sp. nucleopolyhedrovirus (HespNPV), is capable of inhibiting hemolymph melanization and phenoloxidase activity in the host. Intracellular overexpression of this protein substantially enhances viral progeny propagation [48]. Teratocyte-specific CvT-serpins of *Cotesia vestalis* have undergone gene duplication and positive selection, exerting diversified roles in suppressing host melanization, inhibiting bacteria and regulating nutrient metabolism to mediate host immune and metabolic homeostasis [49], while two venom serpins (MmvSPN-1 and MmvSPN-2) from *Microplitis mediator* target host clip-domain serine protease HacSP29 to repress prophenoloxidase activation and antimicrobial peptide production [50]. Combined with previous findings, our results indicate that multiple serpin proteins of *N. bombycis* participate in the inhibition of host hemolymph melanization.

Subcellular localization can provide important functional clues for microsporidian proteins, as several validated effectors exhibit organelle-specific activities, for example, the nucleus-targeted effector EnP1 of microsporidia enters the host nucleus and alters chromatin or epigenetic states, linking nuclear localization to host gene regulation [51,52]. Some host-interacting serpin proteins exhibit stage-dependent localization patterns, suggesting that localization may reflect dynamic roles during proliferation and sporogony, as seen with NbSPN14, which initially localized in the cytosol and subsequently translocated to the host nucleus, inhibiting the host’s apoptotic ability via the caspase BmICE [13]. NbSPN4 was localized to the nucleus, suggesting that NbSPN4 may have functions distinct from melanization suppression, including potential roles in nuclear processes in the host.

Microsporidia deploy secreted effector proteins to systematically reprogram host cellular functions by modulating multiple signaling pathways, including epigenetic regulation, energy metabolism, apoptosis, protein degradation, immune responses, and cell cycle progression [15,53,54,55]. Furthermore, microsporidian infection substantially remodels the composition of host commensal microbiota, thereby triggering diverse physiological abnormalities and even host mortality [56,57]. Mechanistically, microsporidia indirectly reshape the interplay between hosts and their commensal microorganisms by modulating host innate immune responses and altering tissue morphological characteristics [58]. Conversely, host commensal microbiota can be exploited to establish effective strategies for the prevention and control of microsporidian infection. The study has shown that genetically modifying the honeybee gut symbiont *Snodgrassella alvi* can activate honeybee oxidative immunity and effectively inhibit the proliferation of *Nosema ceranae* [59]. Furthermore, nutritional stress increases the susceptibility of various hosts to microsporidian infection [57]. For instance, the fitness benefits conferred by microsporidia on *Anopheles arabiensis* are diet-dependent [60], indicating that nutritional regulation can serve as a general strategy for the prevention and control of microsporidiosis.

Microsporidians possess highly reduced genomes with extensive gene loss. Notably, the serpin gene family has undergone expansion in *N. bombycis*. Therefore, it is reasonable to propose that serpins are key mediators of host–parasite interactions between *N. bombycis* and the silkworm, underscoring the need to systematically identify NbSPNs that modulate host immune responses, define their molecular functions, and determine their cognate target proteases. Such efforts, combined with well-designed in vivo validation studies, will provide a clearer mechanistic understanding of NbSPN-driven immune regulation and help clarify their contributions to parasite survival and pathogenicity.

## 5. Conclusions

Overall, our findings provide a basis for understanding the potential roles of NbSPN4 and NbSPN5 in host–pathogen interactions and immune modulation. Both proteins inhibited hemolymph melanization and suppressed PO activity in vitro, indicating that they can act as immune suppressors and important virulence factors during microsporidian infection. Future studies should aim to identify their cognate target proteases and elucidate how these interactions influence disease intensity and development in vivo. Gaining these insights will deepen our understanding of serpin-mediated immune modulation and may lead to novel strategies for controlling pébrine disease in sericulture.

## Figures and Tables

**Table 1:** List of primers used in this study.

Primers’ Names	Sequence (5′-3′)	Amplicon Size
PD2P-NbSPN4-F	CCACCACGGTTCTGGTGGATCCATGGAAGTACAAGAAGAACACG	1089 bp
PD2P-NbSPN4-R	CTTTACTTACTTATTAGTGTTAACCTAAATATTTGGTAATAAAGAG	-
PD2P-NbSPN5-F	CCACCACGGTTCTGGTGGATCCATGGATTTTCTTGAAATTTCAA	1050 bp
PD2P-NbSPN5-R	CTTTACTTACTTATTAGTGTTATTTTTCAGCTTGACCAATACAA	-
PD2P-F	TAACACTAATAAGTAAGTAAAG	5357 bp
PD2P-R	GGATCCACCAGAACCGTGGTGG	-
Nb-SPN-4-Epi-28a-F	GCAAATGGGTCGCGGATCCATGGAAGTACAAGAAGAACACG	632 bp
Nb-SPN-4-Epi-28a-R	CGAGTGCGGCCGCAAGCTTCGAGGTTTTATTGCTTTCAATATTATCA	-
Nb-SPN-5-Epi-28a-F	GCAAATGGGTCGCGGATCCATGGATTTTCTTGAAATTTCAAAAACC	623 bp
Nb-SPN-5-Epi-28a-R	CGAGTGCGGCCGCAAGCTTACTGTAATCATTTCCATTTCTAGTGATATAC	-

## Data Availability

The original contributions presented in this study are included in the article/Supplementary Material. Further inquiries can be directed to the corresponding authors.

## Supplementary Materials

The following supporting information can be downloaded at: https://www.mdpi.com/article/10.3390/microorganisms14061254/s1, Figure S1: Predicted subcellular localization and transmembrane topology of NbSPN4 and NbSPN5.
