# Supplementary material for: Serpin 4/5 of Nosema bombycis: Molecular Characterization, Subcellular Localization and Pathogenic Roles in Interactions with Bombyx mori
Source: Microorganisms. 2026 Jun 2;14(6):1254. doi: 10.3390/microorganisms14061254 (PMC13303414; doi:10.3390/microorganisms14061254)
Supplement: Supplementary file 1 [file microorganisms-14-01254-s001.zip › Figure S1.pdf]

Figure S1

Predicted subcellular localization and transmembrane topology of NbSPN4 and NbSPN5.

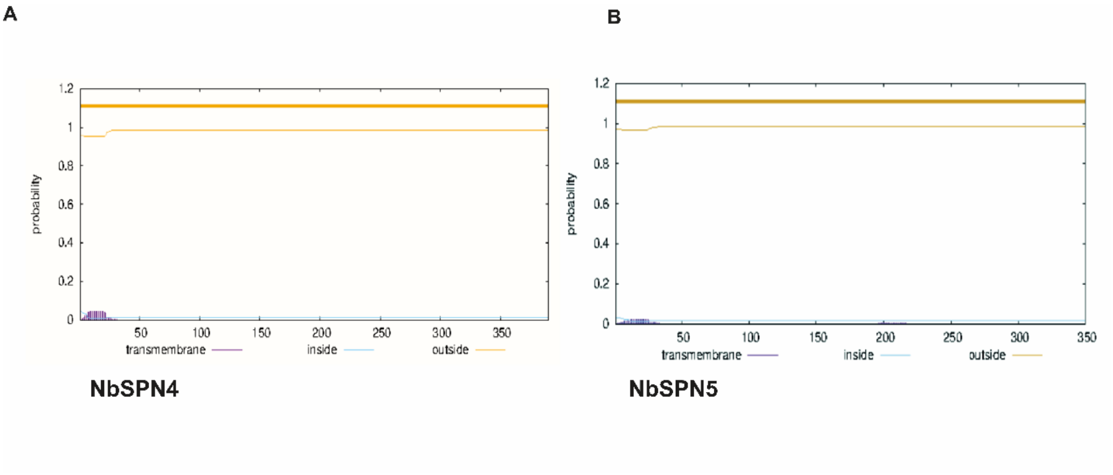

Figure S1. Predicted subcellular localization and transmembrane topology of NbSPN4 and NbSPN5. (A) NbSPN4 and (B) NbSPN5 were analyzed for membrane topology and subcellular localization. The x-axis represents amino-acid position, and the y-axis indicates the predicted probability. Purple bars denote predicted transmembrane regions, the blue line indicates cytoplasmic (inside) localization probability, and the yellow line indicates extracellular (outside) localization probability.
